# Clinical perspectives on post-cholecystectomy syndrome: a narrative review

**DOI:** 10.1080/07853890.2025.2496408

**Published:** 2025-04-30

**Authors:** Changjin Nam, Jun Suh Lee, Ji Su Kim, Tae Yoon Lee, Young Chul Yoon

**Affiliations:** a Kyungpook National University Medical College, Daegu, Republic of Korea; b Department of Surgery, Bucheon Sejong Hospital, Bucheon, Republic of Korea; c Department of Surgery, Incheon St. Mary’s Hospital, Incheon, Republic of Korea

**Keywords:** Cholecystectomy, diagnostic criteria, gut microbiota alterations, sphincter of Oddi dysfunction, treatment outcomes

## Abstract

**Introduction:**

Post-cholecystectomy syndrome (PCS) is a complex condition characterized by persistent or new symptoms following gallbladder removal, affecting up to 47% of patients. Despite being recognized since 1947, there is still no consensus on its etiology, diagnosis, and treatment.

**Areas covered:**

This narrative review explores the multifactorial etiology of PCS, including biliary and extra-biliary factors, and its varied clinical manifestations. A systematic literature search was conducted using keywords like ‘etiology’, ‘clinical manifestations’, ‘diagnostic challenges’, and ‘management strategies’. The review covers traditional diagnostic methods, recent insights into pathophysiology, and current management approaches, such as dietary modifications, pharmacological treatments, and endoscopic interventions, with a focus on patient selection.

**Expert opinion:**

PCS presents significant clinical challenges due to its diverse presentations and lack of standardized diagnostic and therapeutic protocols. Effective management starts with careful patient selection before cholecystectomy to prevent unnecessary surgeries and reduce postoperative complications. Future research should aim to refine diagnostic criteria and develop predictive models for identifying at-risk patients. Personalized management strategies incorporating genetic, biological, and clinical factors are essential for improving outcomes. An integrated, patient-centered approach is crucial for addressing PCS complexities and enhancing the quality of life for affected patients.

## ARTICLE HIGHLIGHTS

- Importance of patient selection: Careful patient selection before cholecystectomy is essential to reduce the risk of post-cholecystectomy syndrome (PCS) and improve surgical outcomes.
- Complexity of PCS: PCS affects up to 47% of patients and involves a multifactorial etiology including biliary and extra-biliary factors, gut microbiota changes, and pain sensitization.
- Diagnostic challenges: Accurate diagnosis of PCS is complicated by its diverse clinical manifestations. Advanced diagnostic tools such as Endoscopic Ultrasound (EUS) and SeHCAT are enhancing diagnostic precision, particularly in identifying remnant stones and bile acid diarrhea.
- Management strategies: Effective management requires a multidisciplinary approach incorporating dietary modifications, pharmacological treatments, and endoscopic interventions tailored to individual patient profiles.
- Predictive factors: Early identification of predictive factors for chronic pain and functional gastrointestinal disorders is crucial for preventing PCS and enhancing patient quality of life.
- Future research directions: Focus on developing standardized diagnostic criteria, refining patient selection processes, and exploring personalized treatment strategies to achieve optimal patient outcomes.

## 1. Introduction

Post-cholecystectomy syndrome (PCS) represents a complex spectrum of gastrointestinal symptoms persisting or emerging post-cholecystectomy, affecting 5–47% of patients [1–5]. First described in 1947 by Womack and Crider, the etiology of PCS is multifaceted, encompassing biliary, extra-biliary organic, and functional factors [5,6]. Recent advances have highlighted the influence of gut microbiota and dietary patterns on PCS development, alongside traditional considerations like pain sensitization [7,8]. Diagnosing PCS poses unique challenges due to its diverse clinical manifestations and underlying causes. The role of specific diagnostic tests like 75-Selenium Homotaurocholic Acid Test (SeHCAT) and endoscopic retrograde cholangiopancreatography (ERCP) has been emphasized in recent research [9,10]. In terms of management, dietary modifications and targeted medication are pivotal [8,11]. Surgical interventions also play a critical role, especially for complications such as bile duct injuries and strictures.

The clinical relevance of PCS is significant, considering the steady increase in cholecystectomy cases. However, a consensus is lacking in all aspects of the syndrome, from diagnosis to treatment. By integrating historical perspectives with recent research, this review seeks to provide a comprehensive understanding of PCS, encompassing its etiology, clinical manifestations, and management strategies.

## 2. Etiology and pathophysiology

### 2.1. Traditional understanding of PCS

Traditional understanding of PCS encompasses a variety of causes. Up to 34% of patients still suffer from some type of abdominal pain after cholecystectomy, often without any underlying condition [12]. Beyond this initial period, the most common cause of complications shifts, with retained ductal stones in the biliary tree emerging as the predominant issue, affecting 4–40% of participants [13,14]. Sphincter of Oddi dysfunction (SOD) accounts for 1.8–31% of cases in an unselected population with PCS [14–16], and in a population pre-selected for likely SOD, true SOD was confirmed in only 25–47% of cases [17–19] based on criteria such as liver biochemistry, ERCP, or manometry [20–22]. However, a significant portion of cases, 4.1–50%, remain without a clear etiological cause [13,17–19].

### 2.2. Recent developments

#### 2.2.1. Impact of gut microbiota alterations post-cholecystectomy and role of dietary patterns

Recent studies have emphasized the significant impact of cholecystectomy on gut microbiota. An experimental study meta-analyzing 218 raw 16S rRNA gene sequencing datasets reveals major alterations in gut microbiota structure and function due to cholecystectomy, with dietary patterns playing a crucial role in these changes [7]. Similarly, a case-control study with 129 participants associates PCS in female patients with gut microbiota particularities, alongside older age, lifestyle factors, and medical history [23]. These findings suggest a strong correlation between gut dysbiosis, unbalanced Firmicutes/Bacteroidetes (F/B) ratios, and abdominal complaints in PCS.

To summarize the findings of these studies, the evidence suggests that cholecystectomy significantly alters gut microbiota, contributing to PCS, with disruptions in the F/B ratio potentially leading to bile acid dysregulation and gastrointestinal symptoms.

#### 2.2.2. Predictive factors for chronic post-cholecystectomy pain

Emerging research also points to the role of early postoperative pain intensity in chronic post-cholecystectomy pain. A prospective cohort study with 100 participants concluded that the visceral pain response during the first postoperative week can predict the risk of chronic pain after laparoscopic cholecystectomy [24]. This study specifically measured various types of pain during the first postoperative week, including overall pain, incisional pain (somatic pain component), deep abdominal pain (visceral pain component), and shoulder pain (referred pain component) using a 100-mm visual analogue scale. The cumulative visceral pain score during the first week and the number of preoperative biliary pain attacks were found to be independent risk factors for developing chronic unexplained pain 12 months postoperatively. This indicates that intense visceral pain shortly after surgery is a critical predictor of long-term pain outcomes.

Another prospective cohort study with 150 patients shows that significant chronic pain post-laparoscopic cholecystectomy is relatively low but closely associated with the intensity of acute postoperative pain [4]. This study conducted a comprehensive analysis involving preoperative clinical data, cold pressor test, state of neuroticism, and early postoperative pain intensity. Their findings revealed that 20 patients reported moderate or severe chronic pain one year after surgery. Notably, these chronic pain patients had significantly more intense acute postoperative pain compared to those without chronic pain. This study highlights the association between acute postoperative pain and the development of chronic pain.

## 3. Clinical manifestations

PCS encompasses a wide array of gastrointestinal and systemic symptoms that persist or emerge following cholecystectomy. While cholecystectomy effectively resolves biliary pain, it may contribute to or fail to alleviate other gastrointestinal symptoms, underscoring the complexity of PCS. PCS is variably defined in the literature, with some studies including persistent preoperative symptoms, while others focus on new or worsening symptoms postoperatively [1,25–27]. Distinguishing between residual symptoms from preexisting conditions (e.g. FGIDs, SOD) and true PCS is crucial.

Several gastrointestinal disorders, such as FGIDs, gastroparesis, and common bile duct stones, can be diagnosed preoperatively. Identifying these conditions before surgery is essential, as misattribution of symptoms to gallbladder disease alone may lead to persistent postoperative symptoms. A thorough preoperative evaluation, including consideration of EUS and ERCP when necessary, may improve patient selection and postoperative outcomes.

Here are some specific conditions associated with PCS.

### 3.1. Functional gastrointestinal disorders (FGIDs)

The relationship between cholecystectomy and FGIDs has been a point of debate. A prospective study involving patients with symptomatic gallstone disease revealed a significant overlap of FGIDs before and after cholecystectomy [28]. Notably, while some preoperative FGID symptoms improved post-surgery, the incidence of new-onset FGIDs, including functional dyspepsia and functional diarrhea, increased, indicating a need for careful pre- and postoperative evaluation of FGID symptoms in these patients.

Furthermore, a prospective observational study investigates the prevalence of functional dyspepsia (FD) and irritable bowel syndrome (IBS) in patients undergoing cholecystectomy [29]. Their findings highlighted that one-third of patients eligible for cholecystectomy met the criteria for FD/IBS, with a significant portion experiencing persisting nonbiliary abdominal pain post-surgery. This underscores the importance of considering coexisting FD/IBS in the management of patients undergoing cholecystectomy.

### 3.2. Bile acid diarrhea and related disorders

The diagnosis and management of Bile Acid Diarrhea (BAD) post-cholecystectomy is crucial. A survey of UK experts identified the SeHCAT as the diagnostic standard for assessing BAD, reflecting its significance in understanding and managing this condition [30]. The recognition of BAD as a prevalent issue among post-cholecystectomy patients underscores the importance of awareness and consideration of this condition in the overall PCS management strategy.

#### 3.2.1. Gastroparesis in diabetic patients post-cholecystectomy

The incidence of gastroparesis following laparoscopic cholecystectomy, particularly in diabetic patients, has been reported [31]. This study identified factors such as the duration of diabetes and patient age as significant predictors of gastroparesis development post-cholecystectomy, highlighting the need for special attention to diabetic patients undergoing this procedure.

PCS presents a complex challenge requiring a multidisciplinary approach to management. The wide spectrum of symptoms, ranging from RUQ pain and diarrhea to FGIDs and BAD, necessitates a comprehensive evaluation and individualized treatment strategies. Future research should focus on identifying predictive factors for PCS and developing guidelines for the management of this condition, ensuring improved patient outcomes post-cholecystectomy.

To understand the spectrum of definitions, etiologies, and symptoms associated with PCS, we present a summary in Table 1. This table compiles key studies and provides a comparative overview of the varying factors contributing to PCS.

**Table 1.** Definition, etiology, and symptoms of post cholecystectomy syndrome (PCS).

Reference	Definition	Etiology	Symptoms
Topazian et al., 2004 [32]	Chronic abdominal pain after cholecystectomy	Sphincter of Oddi dysfunction (SOD)	Chronic abdominal pain
Druart-Blazy et al., 2005 [13]	Pain, abnormal liver tests post-cholecystectomy	SOD	Biliary-like pain
Madácsy et al., 2006 [18]	Need for ERCP due to SOD after cholecystectomy	SOD	Biliary-like pain
Filip et al., 2009 [16]	Abdominal pain, jaundice, or dyspepsia within 3 years	Common bile duct stones, chronic pancreatitis, SOD	Abdominal pain, jaundice, dyspepsia
Blichfeldt-Eckhardt et al., 2014 [24]	Chronic pain after laparoscopic cholecystectomy	SOD, bile duct stone, visceral hyperalgesia, central sensitization	Chronic pain
Altomare et al., 2017 [11]	Nausea, bloating, diarrhea, abdominal pain	Nutritional/metabolic factors	Diarrhea, bloating, abdominal pain
Shin et al., 2018 [8]	Abdominal pain, dyspepsia, constipation, diarrhea	Dietary factors (high animal protein, cholesterol, low vegetables)	Abdominal pain, dyspepsia, diarrhea
Georgescu et al., 2022 [23]	Symptoms similar to pre-cholecystectomy	Gut dysbiosis, age, sedentary lifestyle, diabetes	Abdominal pain, bloating, bowel changes

## 4. Diagnostic challenges

The diagnosis of PCS presents unique challenges due to its varied clinical manifestations and underlying causes. Recent studies have highlighted several diagnostic approaches and their utility in identifying PCS.

Endoscopic Retrograde Cholangiopancreatography (ERCP) is a key diagnostic and therapeutic tool for PCS, particularly in cases of choledocholithiasis, papillary inflammatory stricture, and Sphincter of Oddi dysfunction. While effective in clarifying causes and guiding treatment, ERCP should be used judiciously due to its potential complications.

Endoscopic Ultrasound (EUS) is valuable for detecting remnant stones in the cystic duct or bile duct that may be missed by conventional imaging. Studies have shown that an algorithmic approach incorporating EUS reduces unnecessary ERCP procedures, lowering morbidity and improving diagnostic precision. This approach typically begins with liver function tests (LFTs) and transabdominal ultrasound (TUS). If common bile duct (CBD) dilation (≥10 mm) or abnormal LFTs are present, EUS is recommended to confirm abnormalities before ERCP is considered [16].

**Figure 1.**
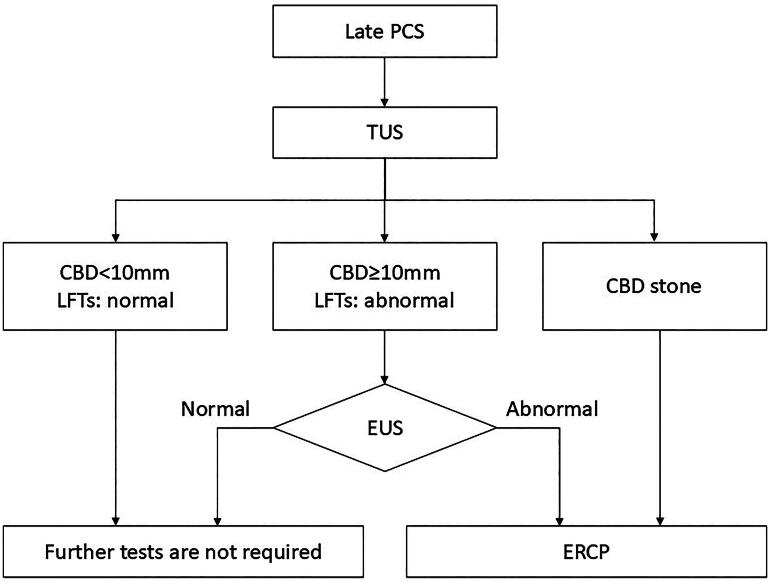
Algorithm for diagnosis of late post cholecystectomy syndrome (PCS). An algorithmic approach to the diagnosis of late PCS as reported by Filip et al. (J Gastrointestin Liver Dis. 2009 Mar;18(1):67–71.). TUS transabdominal Ultrasound; CBD common bile duct; LFT liver function test; EUS endoscopic ultrasound; ERCP endoscopic retrograde cholangiopancreatography.

Figure 1 illustrates the diagnostic algorithm for late PCS, outlining a structured approach to imaging and intervention.

For bile acid diarrhea, SeHCAT remains a key diagnostic tool, though accessibility limitations exist. Alternative biomarkers, including 7α-hydroxy-4-cholesten-3-one (C4) and fibroblast growth factor 19 (FGF19), have shown promise in diagnosing bile acid diarrhea more efficiently [10,30,33].

Emerging research suggests that gut microbiome analysis may provide insight into PCS pathophysiology. Alterations in microbiome composition, particularly increased Proteobacteria, have been linked to persistent gastrointestinal symptoms. However, further studies are needed to validate microbiome-based diagnostics in PCS [7].

Cost-effectiveness considerations are important when selecting diagnostic strategies for PCS, yet evidence in this area remains limited. One study evaluating the cost-effectiveness of ERCP, EUS, and MRCP found that ERCP is the most cost-effective option for patients with a high probability of requiring therapeutic intervention [34]. However, given the potential risks associated with ERCP, including post-ERCP pancreatitis, the balance between cost and patient safety must be carefully considered. Further research is needed to comprehensively assess the cost-effectiveness of various diagnostic and therapeutic approaches for PCS.

A comprehensive diagnostic approach—integrating ERCP, EUS, and biochemical markers—is essential for accurately identifying PCS and tailoring appropriate management strategies.

## 5. Management and treatment strategies

### 5.1. Conventional approaches

#### 5.1.1. Dietary management

Dietary modifications play an important role in managing PCS symptoms. Studies suggest that a high-fat, high-cholesterol diet post-cholecystectomy can exacerbate bile acid disturbances and gut microbiome dysbiosis, increasing intestinal inflammation [7]. Conversely, a low-fat, high-fiber diet may help alleviate symptoms [8,11].

#### 5.1.2. Medical management

Cisapride has been shown to enhance biliary drainage in postcholecystectomy syndrome (PCS) patients, as demonstrated in a randomized, placebo-controlled crossover trial involving 19 female participants. The study found that cisapride significantly hastened biliary emptying, with a shorter median half emptying time compared to placebo [35]. However, cisapride was associated with increased symptom severity, particularly in patients whose PCS symptoms resembled biliary pain induced during ERCP contrast injection. These findings suggest that while cisapride may improve bile flow, its use in PCS is limited by its potential to exacerbate biliary-type discomfort.

For sphincter of Oddi dysfunction (SOD), calcium channel blockers such as nifedipine have shown efficacy in reducing pain episodes [14,36]. Additionally, endoscopic botulinum toxin injections have demonstrated short-term symptom relief and may help predict response to sphincterotomy [37]. Alternative pharmacologic options such as glyceryl trinitrate (GTN) have also been considered, though further research is needed to establish their long-term effectiveness [38].

Opiate use has been associated with worsening PCS symptoms, particularly in younger patients with a narrow bile duct. Reducing opiate exposure before and after cholecystectomy may improve postoperative symptom outcomes [13].

For bile acid diarrhea, bile acid sequestrants such as cholestyramine have been effective, particularly in patients with confirmed bile acid malabsorption via SeHCAT scanning [33].

#### 5.1.3. Endoscopic management

Endoscopic interventions are crucial for managing PCS cases related to SOD and bile duct abnormalities. Studies have explored the efficacy of sphincterotomy, with mixed results. While early research suggested benefit, the EPISOD trial found no significant reduction in pain-related disability compared to a sham procedure, prompting reconsideration of its routine use [39,40].

A significant advancement in PCS treatment involves the management of remnant cystic duct lithiasis (RCDL). Cholangioscopy-guided electrohydraulic lithotripsy has emerged as a minimally invasive alternative for patients in whom conventional ERCP fails [41]. This technique offers a promising alternative to surgery, which carries risks of bile duct injury and infection.

Recent advancements in endoscopic technology have expanded treatment options for post-cholecystectomy Mirizzi syndrome, traditionally managed with surgery. A novel endoscopic approach has been successfully used to remove residual cystic duct stones, offering a minimally invasive alternative [42,43]. These innovations highlight the growing role of endoscopy in managing complex biliary conditions.

Surgical excision remains necessary in cases where endoscopic treatment is insufficient, as highlighted by a retrospective study in which 50% of RCDL patients required surgical intervention [44]. These findings underscore the need for individualized treatment plans based on patient-specific factors.

Table 2 categorizes treatment strategies by indication and outlines therapeutic interventions for PCS.

**Table 2.** Treatments of post cholecystectomy syndrome (PCS).

Category	Reference	Indication	Treatment	Key findings
Dietary management	Altomare et al., 2017 [11]; Shin et al., 2018 [8]	Alimentary disorders	Low-fat, high-fiber diet	Helps manage post-cholecystectomy symptoms
Medical management	Farup et al., 1991 [35]	Biliary drainage issues	Cisapride (20 mg twice/day)	Improves drainage but may worsen symptoms
	Khuroo et al., 1992 [14]	SOD	Nifedipine (30–60 mg/day)	Reduces pain episodes
	Sand et al., 2005 [36]	SOD	Calcium Channel Blockers	Inhibits sphincter contractions
	Staritz et al., 1985 [38]	SOD	Glyceryl trinitrate (1.2 mg)	Relaxes sphincter, useful for biliary colic
	Wehrmann et al., 1998 [37]	SOD	Botulinum toxin injection	Temporary symptom relief
	Pauls et al., 2016 [45]	SOD	Duloxetine (60 mg/day)	Effective but high dropout rate due to side effects
	Okoro et al., 2008 [46]	Bile microlithiasis	Ursodeoxycholic acid (300 mg twice/day)	Relieves symptoms
	Borghede et al., 2011 [33]	Chronic diarrhea	Cholestyramine	Effective in symptom relief
Endoscopic management	Cicala et al., 2002 [52]; Cotton et al., 2014, 2018 [39,40]	SOD	Endoscopic sphincterotomy	Effective in select cases, but controversial after EPISOD trial

### 5.2. Emerging treatments and interventions

#### 5.2.1. Acupuncture and related therapies

Recent studies have explored the role of acupuncture in managing PCS. A systematic review and meta-analysis encompassing 14 RCTs with 1593 participants evaluated the efficacy of acupuncture combined with conventional medicine (CM) [47]. The meta-analysis found that while acupuncture combined with CM did not significantly reduce pain compared to CM alone, it did significantly reduce the incidence of postoperative nausea and vomiting (PONV) and improved gastrointestinal function recovery. Additionally, acupuncture combined with traditional Chinese medicine and CM, as well as acupuncture as monotherapy, showed potential in improving gastrointestinal function with acceptable adverse events.

#### 5.2.2. Interventional pathways

In addressing the challenging condition of PCS, a six-year prospective audit offers critical insights into innovative interventional pathways [48]. The audit, involving 60 patients, underscores the significant prevalence of abdominal myofascial pain syndrome—a somatic pathology marked by trigger points in the abdominal musculature, often overlooked as a contributor to persistent post-cholecystectomy discomfort. The study’s intervention pathway, comprising abdominal plane blocks and epigastric port site trigger injections with steroids, followed by pulsed radiofrequency treatment for non-responders, demonstrates a considerable impact in alleviating pain, reducing opioid consumption, and decreasing emergency visits. This nuanced approach to managing abdominal myofascial pain syndrome reveals the necessity for a broader diagnostic lens and diversified treatment modalities in post-cholecystectomy care, challenging the traditional focus on visceral etiologies.

The study concluded that abdominal myofascial pain syndrome is a poorly recognized cause of PCS. By implementing a structured interventional management pathway, significant benefits were observed in terms of pain relief, patient satisfaction, and reduced healthcare utilization. This highlights the importance of considering somatic as well as visceral causes when managing PCS and suggests that a multidisciplinary approach can significantly improve patient outcomes.

### 5.3. Preoperative considerations for cholecystectomy to prevent PCS

In managing PCS, it’s essential to clarify patient profiles and pinpoint risk factors. Recent trials have shed light on these areas, aiding in a more personalized approach to cholecystectomy.

The SECURE trial was a large-scale, multicenter, randomized, parallel-arm study that compared a restrictive cholecystectomy strategy with usual care to see if a more conservative approach could reduce unnecessary surgeries without compromising patient outcomes [49]. The study included 1067 patients who had abdominal pain and ultrasound-confirmed gallstones or sludge. Participants were randomly assigned to either an usual care group or a restrictive strategy group, where cholecystectomy was recommended only if patients met five specific criteria: severe pain episodes, pain lasting 15–30 min or more, pain located in the epigastrium or right upper quadrant, pain radiating to the back, and a positive pain response to simple analgesics. After 12 months, 56% of patients in the restrictive strategy group were pain-free compared to 60% in the usual care group (P_non-inferiority = 0.316). The restrictive strategy also resulted in fewer cholecystectomies (68 vs. 75%; *p* = 0.01) without a significant increase in complications, suggesting that many surgeries could be avoided without affecting patient safety. Additionally, at the 5-year follow-up, the results showed that 62.8% of patients in the usual care group were pain-free compared to 61.2% in the restrictive strategy group (non-inferiority *p* = 0.18), demonstrating no significant long-term differences in pain relief between the two groups [50]. The restrictive strategy was associated with a lower cholecystectomy rate (73.2 vs. 81.5%, *p* = 0.001), without an increase in biliary or surgical complications.

The combined efforts of the SECURE and SUCCESS trials aimed to create and validate a multivariable prediction model to better select patients for surgery [51]. This model considered patient characteristics, comorbidities, surgical outcomes, and pain/symptoms at baseline and six months follow-up. Key predictors of pain reduction included older age, no prior abdominal surgery, higher baseline pain scores, pain radiation to the back, pain relief with simple analgesics, presence of nausea, and absence of heartburn. The model demonstrated strong predictive ability (C statistic, 0.74; 95% CI, 0.70–0.78) and fair calibration, emphasizing the significance of these factors in forecasting pain relief after cholecystectomy.

Additionally, the PERFECT trial focused on determining the prevalence of FD and IBS in patients eligible for cholecystectomy and examining their impact on postoperative outcomes [29]. This study involved 401 patients with gallstones and used Rome IV criteria to assess FD/IBS prevalence. Results showed that 34.9% of patients met the criteria for FD/IBS. After cholecystectomy, only 40.7% of patients with FD/IBS were pain-free compared to 64.4% of those without FD/IBS (*p* < 0.001). These findings highlight the need to consider FD and IBS in preoperative assessments, as they are significantly linked to continued postoperative pain.

Collectively, these studies advocate for a more judicious approach to cholecystectomy, emphasizing the importance of thorough preoperative evaluations that include assessments for FD and IBS. This approach aligns surgical interventions with the specific needs of each patient, aiming to improve overall outcomes through more individualized and evidence-based decision-making.

Preoperative considerations are crucial in preventing PCS, as outlined by recent studies. Table 3 provides a summary of assessment criteria and indicators for surgery based on key trials, helping to identify patients who would benefit most from cholecystectomy.

**Table 3.** Preoperative considerations for cholecystectomy to prevent post cholecystectomy syndrome (PCS).

Reference	Assessment criteria	Indicators for surgery	Details
SECURE Trial (van Dijk et al., 2019) [49]	Age	>50 Years	Older patients are more likely to benefit.
	Pain characteristics	Must meet all criteria	Severe pain attacks, pain in epigastrium or right upper quadrant, pain lasting 15–30 minutes or longer, pain radiating to the back, positive pain response to simple analgesics.
SECURE/Success Trial (Latenstein et al., 2021) [51]	History of abdominal surgery	No prior abdominal surgery	Absence indicates higher probability of pain reduction.
	Nausea	Presence	Presence of nausea is associated with better postoperative outcomes.
	Heartburn	Absence	Absence of heartburn is associated with better postoperative outcomes.
PERFECT Trial (De Jong et al., 2022) [29]	FD/IBS criteria	Absence of FD/IBS	Assess for functional dyspepsia (FD) and irritable bowel syndrome (IBS) using Rome IV criteria.

## 6. Controversies and areas of debate: Sphincter of Oddi dysfunction and its role in PCS

The role of SOD in PCS remains a topic of considerable debate. A review highlights that patients with Sphincter of Oddi stenosis often benefit from sphincterotomy and those with classic biliary pain and evidence of biliary obstruction may have SOD, warranting endoscopic evaluation and therapy. However, it advises against ERCP in patients with atypical post-cholecystectomy pain without clear signs of biliary obstruction [9].

The relationship between psychosocial distress and SOD is also contentious. A retrospective cohort study suggests that a motility disturbance related to psychosocial distress might explain SO dyskinesia in some postcholecystectomy patients [17].

Another study indicates that pain characteristics can differ significantly in PCS patients with and without SOD, with those having definite SOD showing ­symptomatic improvement after endoscopic sphincterotomy (EST), yet many did not become completely pain-free [18].

The use of opiates and its potential role in misdiagnosing SOD has been highlighted in a study, indicating the need for careful history taking to avoid unnecessary procedures [13]. Quantitative choledochoscintigraphy has been suggested as an useful non-invasive diagnostic tool for SOD, as well as a predictor of sphincterotomy outcome [52]. The significance of bile duct crystals in SOD has also been questioned, with a study suggesting they do not contribute significantly to the dysfunction [19].

## 7. Case reports and rare conditions

### 7.1. Remnant gallbladder and biliary pancreatitis

The presence of a remnant gallbladder can lead to post-cholecystectomy syndrome and complications such as biliary pancreatitis. A case reported in 2014 discussed symptomatic calculi in a remnant gallbladder as a rare but significant cause of such ­conditions [53].

Another study, conducted in 1991, evaluated the role of the cystic duct stump in post-cholecystectomy symptoms. The study concluded that the cystic duct stump is seldom a cause for recurrent symptoms by itself and that total excision of the cystic duct does not necessarily prevent postcholecystectomy symptoms [54].

These case reports and studies highlight the importance of considering rare conditions and anatomical anomalies in the diagnosis and management of post-cholecystectomy complications. They underscore the complexity of symptoms that can arise after cholecystectomy and the necessity for thorough evaluation and individualized treatment approaches.

## 8. Conclusion

Post-cholecystectomy syndrome (PCS) remains a significant challenge in clinical practice due to its diverse etiologies and overlapping symptomatology with other gastrointestinal disorders. Effective management requires a structured approach encompassing preoperative assessment, standardized diagnostics, evidence-based interventions, and long-term follow-up strategies.

### 8.1. Preoperative strategies to prevent PCS

The risk of PCS can be mitigated through careful patient selection and preoperative optimization. The SECURE trial and other studies have established clear indicators for surgery, which should guide clinical decision-making (Table 3).

Key recommendations for clinicians before cholecystectomy include:

- Assess for functional gastrointestinal disorders (FGIDs): Utilize Rome IV criteria to screen for functional dyspepsia (FD) and irritable bowel syndrome (IBS). Patients with these conditions have poorer post-cholecystectomy outcomes and may benefit from non-surgical management (PERFECT trial).
- Use validated pain criteria: Ensure patients meet all SECURE trial criteria for biliary-type pain (i.e. epigastric/RUQ pain, severe intensity, lasting 15–30 min, radiating to the back, and responsive to simple analgesics).
- Consider age and surgical history: Patients >50 years and those without prior abdominal surgery have better pain resolution post-cholecystectomy (SUCCESS trial).
- Optimize dietary and metabolic factors: Patients with poor dietary habits (high cholesterol/low fiber intake) should undergo nutritional counseling before surgery, as these factors contribute to PCS (Shin et al.).

### 8.2. Diagnostic strategies for PCS

The heterogeneity of PCS symptoms necessitates a structured diagnostic algorithm (Figure 1). Key steps for clinicians include:

- Initial workup: Perform transabdominal ultrasound (TUS) and liver function tests (LFTs) to identify structural abnormalities (e.g. bile duct dilation, stones).
- Endoscopic Ultrasound (EUS): Recommended if LFTs or TUS suggest common bile duct pathology. EUS can detect remnant cystic duct stones or microlithiasis, which may contribute to PCS.
- SeHCAT scan for bile acid diarrhea (BAD): Chronic diarrhea post-cholecystectomy may indicate bile acid malabsorption. SeHCAT testing and empirical cholestyramine therapy are recommended in such cases (Borghede et al.).
- Manometry for suspected sphincter of Oddi dysfunction (SOD): If biliary-type pain persists without structural abnormalities, consider sphincter of Oddi manometry or botulinum toxin testing before invasive interventions (Wehrmann et al.).

### 8.3. Management of PCS: targeted interventions

PCS management should be symptom-specific and evidence-based (Table 2).

#### 8.3.1. Dietary and pharmacological therapy (first-line approach)

- Bile Acid Diarrhea (BAD): Cholestyramine (Borghede et al.) or newer bile acid binders (e.g. colesevelam)
- FGID-Related PCS Symptoms: Low-fat, high-fiber diets (Altomare et al.)
- Functional Dyspepsia/Postprandial Symptoms: Prokinetics (e.g. domperidone, cisapride) (Farup et al.)
- Chronic Postoperative Pain: Duloxetine 60 mg/day, though high dropout rates due to side effects must be considered (Pauls et al.)

#### 8.3.2. Endoscopic and surgical interventions (for refractory cases)

- Sphincter of Oddi Dysfunction (SOD):
  - Calcium channel blockers (e.g. nifedipine 30–60 mg/day) can reduce sphincter spasms (Khuroo et al.).
  - Botulinum toxin injections may provide temporary relief and predict response to sphincterotomy (Wehrmann et al.).
  - Endoscopic sphincterotomy (EST) remains controversial post-EPISOD trial and should be reserved for documented biliary obstruction (Cotton et al.).
- Remnant Cystic Duct Lithiasis (RCDL):
  - Cholangioscopy-guided electrohydraulic lithotripsy is a viable minimally invasive alternative to surgery (Ryou et al.).
- Refractory Abdominal Myofascial Pain Syndrome:
  - Consider abdominal plane blocks, trigger point steroid injections, or pulsed radiofrequency treatments, as per interventional pathway studies (Lee et al.).

#### 8.3.3. Long-term monitoring and follow-up

Patients with persistent PCS symptoms require structured follow-up protocols:

- Monitor patients postoperatively at 1, 6, and 12 months to assess symptom progression and response to interventions.
- Referral to a multidisciplinary team (gastroenterology, surgery, pain management) for refractory cases.
- Consider gut microbiome testing in patients with recurrent dyspepsia, bloating, or postprandial symptoms, as gut dysbiosis is emerging as a key PCS mechanism (Georgescu et al.).

## 9. Expert opinion

Post-cholecystectomy syndrome (PCS) presents a significant clinical challenge due to its diverse etiologies and complex symptomatology, making the development of effective management strategies difficult. The most crucial factor in the prevention and management of PCS is appropriate patient selection at the preoperative stage. By carefully selecting patients before surgery, unnecessary procedures can be minimized, the risk of PCS can be reduced, and overall quality of life for patients can be improved. According to current research, strengthening preoperative assessments by utilizing predictive factors such as early postoperative pain, history of functional gastrointestinal disorders (FGID), and predisposition to chronic pain can effectively predict the likelihood of PCS development.

For instance, patients with a history of FGID are at a higher risk of persistent or newly emerging symptoms following cholecystectomy. Such patients should be considered for non-surgical treatment options preoperatively, or the necessity of surgery should be reconsidered. This approach helps to prevent unnecessary surgeries, reduce postoperative dissatisfaction, and ultimately enhance patient quality of life.

Despite the clear importance of patient selection, its widespread adoption in clinical practice remains limited due to the lack of standardized diagnostic criteria, predictive models, and the discrepancy between patient expectations and clinical realities. Additionally, traditional surgical approaches are relatively standardized, further hindering individualized patient selection. Therefore, the development of more sophisticated predictive models and patient selection algorithms is urgently needed. Moreover, it is essential to establish educational resources and infrastructure to facilitate the practical application of these models in clinical settings.

Additional research holds significant potential for advancing patient selection for PCS prevention. First, large-scale cohort studies across diverse populations are needed to accurately define high-risk patient groups and develop tailored management strategies for these individuals. Second, new predictive models incorporating genetic factors and gut microbiota changes should be developed to provide more precise risk assessments. Such research would lay the groundwork for improved prevention and management of PCS.

## 10. Future directions and speculative viewpoint

In the near future, preoperative patient selection will become a fundamental component of PCS prevention and management. With the development of advanced predictive models and standardized assessment systems, it will be possible to accurately identify high-risk patients before surgery and develop personalized management strategies based on this information. These advancements will significantly reduce unnecessary surgeries and improve patient outcomes.

Furthermore, research into gut microbiota and other novel areas will deepen our understanding of PCS, enabling the development of more sophisticated, patient-specific management strategies. In the future, the integration of genetic, biological, and clinical data will enable healthcare providers to make more informed decisions regarding the necessity of cholecystectomy and to provide personalized non-surgical treatment or other management options for patients at high risk of PCS. This approach will significantly lower the incidence of PCS and improve patient quality of life.

In conclusion, a personalized approach to patient selection and management will set a new standard for PCS care and ultimately contribute to better outcomes for all patients. With further research and the development of predictive models, the effectiveness of PCS prevention and management will be significantly enhanced, positively impacting both patients and healthcare providers.

## Data Availability

Due to the nature of this narrative review, no original data was used in this manuscript.

## Clinical trial number

This study was not registered, and has no clinical trial number.

## References

[1] Isherwood J, Oakland K, Khanna A. A systematic review of the aetiology and management of post cholecystectomy syndrome. Surgeon. 2019;17(1):33–42. doi: 10.1016/j.surge.2018.04.001. 29730174

[2] Anand AC, Sharma R, Kapur BM, et al. Analysis of symptomatic patients after cholecystectomy: is the term post-cholecystectomy syndrome an anachronism? Trop Gastroenterol. 1995;16:126–131. 8644362

[3] Russello D, Di Stefano A, Scala R, et al. Does cholecystectomy always resolve biliary disease? Minerva Chir. 1997;52(12):1435–1439. 9557456

[4] Bisgaard T, Rosenberg J, Kehlet H. From acute to chronic pain after laparoscopic cholecystectomy: a prospective follow-up analysis. Scand J Gastroenterol. 2005;40(11):1358–1364. doi: 10.1080/00365520510023675. 16334446

[5] Womack NA, Crider RL. The persistence of symptoms following cholecystectomy. Ann Surg. 1947;126(1):31–55. doi: 10.1097/00000658-194707000-00004. 17858976 PMC1803303

[6] Drossman DA. The functional gastrointestinal disorders and the Rome III process. Gastroenterology. 2006;130(5):1377–1390. doi: 10.1053/j.gastro.2006.03.008. 16678553

[7] Xu F, Yu Z, Liu Y, et al. A high-fat, high-cholesterol diet promotes intestinal inflammation by exacerbating gut microbiome dysbiosis and bile acid disorders in cholecystectomy. Nutrients. 2023;15(17):3829. doi: 10.3390/nu15173829. 37686860 PMC10489946

[8] Shin Y, Choi D, Lee KG, et al. Association between dietary intake and postlaparoscopic cholecystectomic symptoms in patients with gallbladder disease. Korean J Intern Med. 2018;33(4):829–836. doi: 10.3904/kjim.2016.223. 29117670 PMC6030420

[9] Tarnasky PR. Post-cholecystectomy syndrome and sphincter of Oddi dysfunction: past, present and future. Expert Rev Gastroenterol Hepatol. 2016;10(12):1359–1372. doi: 10.1080/17474124.2016.1251308. 27762149

[10] Sciarretta G, Furno A, Mazzoni M, et al. Post-cholecystectomy diarrhea: evidence of bile acid malabsorption assessed by SeHCAT test. Am J Gastroenterol. 1992;87(12):1852–1854. 1449156

[11] Altomare DF, Rotelli MT, Palasciano N. Diet After Cholecystectomy. Curr Med Chem. 2019;26(19):3662–3665. doi: 10.2174/0929867324666170518100053. 28521679

[12] Bates T, Ebbs SR, Harrison M, et al. Influence of ­cholecystectomy on symptoms. Br J Surg. 1991;78(8):964–967. doi: 10.1002/bjs.1800780823. 1913118

[13] Druart-Blazy A, Pariente A, Berthelemy P, et al. The underestimated role of opiates in patients with suspected sphincter of Oddi dysfunction after cholecystectomy. Gastroenterol Clin Biol. 2005;29(12):1220–1223. doi: 10.1016/s0399-8320(05)82204-3. 16518275

[14] Khuroo MS, Zargar SA, Yattoo GN. Efficacy of nifedipine therapy in patients with sphincter of Oddi dysfunction: a prospective, double-blind, randomized, placebo-controlled, cross over trial. Br J Clin Pharmacol. 1992;33(5):477–485. doi: 10.1111/j.1365-2125.1992.tb04074.x. 1524959 PMC1381433

[15] Zhou P-H, Liu F-L, Yao L-Q, et al. Endoscopic diagnosis and treatment of post-cholecystectomy syndrome. Hepatobiliary Pancreat Dis Int. 2003;2(1):117–120. 14607662

[16] Filip M, Saftoiu A, Popescu C, et al. Postcholecystectomy syndrome – an algorithmic approach. J Gastrointestin Liver Dis. 2009;18(1):67–71. 19337637

[17] Bennett E, Evans P, Dowsett J, et al. Sphincter of Oddi dysfunction: psychosocial distress correlates with manometric dyskinesia but not stenosis. World J Gastroenterol. 2009;15(48):6080–6085. doi: 10.3748/wjg.15.6080. 20027681 PMC2797665

[18] Madácsy L, Fejes R, Kurucsai G, et al. Characterization of functional biliary pain and dyspeptic symptoms in ­patients with sphincter of Oddi dysfunction: effect of papillotomy. World J Gastroenterol. 2006;12(42):6850–6856. doi: 10.3748/wjg.v12.i42.6850. 17106935 PMC4087441

[19] Quallich LG, Stern MA, Rich M, et al. Bile duct crystals do not contribute to sphincter of Oddi dysfunction. Gastrointest Endosc. 2002;55(2):163–166. doi: 10.1067/mge.2002.121340. 11818916

[20] Longstreth GF, Thompson WG, Chey WD, et al. Functional bowel disorders. Gastroenterology. 2006;130(5):1480–1491. doi: 10.1053/j.gastro.2005.11.061. 16678561

[21] Petersen BT. An evidence-based review of sphincter of Oddi dysfunction: part I, presentations with “objective” biliary findings (types I and II. Gastrointest Endosc. 2004;59(4):525–534. doi: 10.1016/s0016-5107(04)00012-4. 15044889

[22] Petersen BT. Sphincter of Oddi dysfunction, part 2: evidence-based review of the presentations, with ­“objective” pancreatic findings (types I and II) and of presumptive type III. Gastrointest Endosc. 2004;59(6):670–687. doi: 10.1016/s0016-5107(04)00297-4. 15114311

[23] Georgescu D, Caraba A, Ionita I, et al. Dyspepsia and gut microbiota in female patients with postcholecystectomy syndrome. Int J Womens Health. 2022;14:41–56. doi: 10.2147/IJWH.S342882. 35136356 PMC8816732

[24] Blichfeldt-Eckhardt MR, Ording H, Andersen C, et al. Early visceral pain predicts chronic pain after laparoscopic cholecystectomy. Pain. 2014;155(11):2400–2407. doi: 10.1016/j.pain.2014.09.019. 25250720

[25] Jaunoo SS, Mohandas S, Almond LM. Postcholecystectomy syndrome (PCS). Int J Surg. 2010;8(1):15–17. doi: 10.1016/j.ijsu.2009.10.008. 19857610

[26] Kim H, Han IW, Heo JS, et al. Postcholecystectomy syndrome: symptom clusters after laparoscopic cholecystectomy. Ann Surg Treat Res. 2018;95(3):135–140. doi: 10.4174/astr.2018.95.3.135. 30182019 PMC6121167

[27] Peikoff SS. Post cholecystectomy syndrome. Manit Med Rev. 1946;26:69–73. 21011295

[28] Chang JY, Jung H-K, Moon CM, et al. Development of functional gastrointestinal disorder symptoms following laparoscopic cholecystectomy: a prospective cohort study. Front Med. 2023;10:1248465. doi: 10.3389/fmed.2023.1248465. PMC10587431 37869171

[29] de Jong JJ, Latenstein CSS, Boerma D, et al. Functional dyspepsia and irritable bowel syndrome are highly prevalent in patients with gallstones and are negatively associated with outcomes after cholecystectomy: a prospective, multicenter, observational study (PERFECT – Trial). Ann Surg. 2022;275(6):e766–e772. doi: 10.1097/SLA.0000000000004453. 32889877

[30] Walters JRF, Arasaradnam R, Andreyev HJN, UK Bile Acid Related Diarrhoea Network. UK bile acid related diarrhoea network, diagnosis and management of bile acid diarrhoea: a survey of UK expert opinion and practice. Frontline Gastroenterol. 2020;11(5):358–363. doi: 10.1136/flgastro-2019-101301. 32879719 PMC7447276

[31] Al-Mulhim AS. Gastroparesis post-laparoscopic cholecystectomy in diabetic patients. Updates Surg. 2017;69(1):89–93. doi: 10.1007/s13304-017-0417-0. 28188572

[32] Topazian M, Hong-Curtis J, Li J, et al. Improved predictors of outcome in postcholecystectomy pain. J Clin Gastroenterol. 2004;38(8):692–696. doi: 10.1097/01.mcg.0000135371.03222.df 15319654. 15319654

[33] Borghede MK, Schlütter JM, Agnholt JS, et al. Bile acid malabsorption investigated by selenium-75-homocholic acid taurine ((75)SeHCAT) scans: causes and treatment responses to cholestyramine in 298 patients with chronic watery diarrhoea. Eur J Intern Med. 2011;22(6):e137–e140. doi: 10.1016/j.ejim.2011.08.013. 22075299

[34] Ainsworth AP, Rafaelsen SR, Wamberg PA, et al. Cost-effectiveness of endoscopic ultrasonography, magnetic resonance cholangiopancreatography and endoscopic retrograde cholangiopancreatography in patients ­suspected of pancreaticobiliary disease. Scand J Gastroenterol. 2004;39(6):579–583. doi: 10.1080/00365520410004442. 15223684

[35] Farup PG, Tjora S, Tholfsen JK. Effect of cisapride on symptoms and biliary drainage in patients with postcholecystectomy syndrome. Scand J Gastroenterol. 1991;26(9):945–950. doi: 10.3109/00365529108996247. 1947787

[36] Sand J, Arvola P, Nordback I. Calcium channel antagonists and inhibition of human sphincter of Oddi contractions. Scand J Gastroenterol. 2005;40(12):1394–1397. doi: 10.1080/00365520510023800. 16293553

[37] Wehrmann T, Seifert H, Seipp M, et al. Endoscopic injection of botulinum toxin for biliary sphincter of Oddi dysfunction. Endoscopy. 1998;30(8):702–707. doi: 10.1055/s-2007-1001392. 9865560

[38] Staritz M, Poralla T, Ewe K, et al. Effect of glyceryl trinitrate on the sphincter of Oddi motility and baseline pressure. Gut. 1985;26(2):194–197. doi: 10.1136/gut.26.2.194. 3917965 PMC1432421

[39] Cotton PB, Durkalski V, Romagnuolo J, et al. Effect of endoscopic sphincterotomy for suspected sphincter of Oddi dysfunction on pain-related disability following cholecystectomy: the EPISOD randomized clinical trial. JAMA. 2014;311(20):2101–2109. doi: 10.1001/jama.2014.5220. 24867013 PMC4428324

[40] Cotton PB, Pauls Q, Keith J, et al. The EPISOD study: long-term outcomes. Gastrointest Endosc. 2018;87(1):205–210. doi: 10.1016/j.gie.2017.04.015. 28455162

[41] Ryou SH, Kim HJ. Successful removal of remnant cystic duct stump stone using single-operator cholangioscopy-guided electrohydraulic lithotripsy: two case reports. Clin Endosc. 2023;56(3):375–380. doi: 10.5946/ce.2021.273. 36600660 PMC10244158

[42] Li Y, Zhang L, Hou S. Cystic duct stones in postcholecystectomy Mirizzi syndrome – a novel endoscopic treatment. Rev Esp Enferm Dig. 2022;114(9):557–558. doi: 10.17235/reed.2022.8802/2022. 35360910

[43] Odemis B, Oztaş E, Akpınar MY, et al. An alternative treatment for postcholecystectomy Mirizzi’s syndrome: cystic duct balloon dilation. Endoscopy. 2015;47(S 01):E371–E371. doi: 10.1055/s-0034-1392593. 26273766

[44] Phillips MR, Joseph M, Dellon ES, et al. Surgical and endoscopic management of remnant cystic duct lithiasis after cholecystectomy–a case series. J Gastrointest Surg. 2014;18(7):1278–1283. doi: 10.1007/s11605-014-2530-4. 24810238

[45] Pauls QI, Durkalski-Mauldin V, Brawman-Mintzer O, et al. Duloxetine for the Treatment of Patients with Suspected Sphincter of Oddi Dysfunction: A Pilot Study. Dig Dis Sci. 2016;61(9):2704–2709. doi: 10.1007/s10620-016-4187-1 27165434 27165434

[46] Okoro N, Patel A, Goldstein M, et al. Ursodeoxycholic acid treatment for patients with postcholecystectomy pain and bile microlithiasis. Gastrointest Endosc. 2008;68(1):69–74. doi: 10.1016/j.gie.2007.09.046 18577477 18577477

[47] Yin Z, Xiao Q, Xu G, et al. Acupuncture for the postcholecystectomy syndrome: a systematic review and meta-analysis. Evid Based Complement Alternat Med. 2020;2020(1):7509481. doi: 10.1155/2020/7509481. 32802133 PMC7414376

[48] Lee H, Kukreja Y, Niraj G. Interventional pathway in the management of refractory post cholecystectomy pain (PCP) syndrome: a 6-year prospective audit in 60 patients. Scand J Pain. 2023;23(4):712–719. doi: 10.1515/sjpain-2022-0090. 36779538

[49] van Dijk AH, Wennmacker SZ, de Reuver PR, et al. Restrictive strategy versus usual care for cholecystectomy in patients with gallstones and abdominal pain (SECURE): a multicentre, randomised, parallel-arm, non-inferiority trial. Lancet. 2019;393(10188):2322–2330. doi: 10.1016/S0140-6736(19)30941-9. 31036336

[50] Comes DJ, Wennmacker SZ, Latenstein CSS, et al. Restrictive strategy vs usual care for cholecystectomy in patients with abdominal pain and gallstones: 5-year follow-up of the SECURE randomized clinical trial. JAMA Surg. 2024;159(11):1235–1243. doi: 10.1001/jamasurg.2024.3080. 39167382 PMC11339699

[51] Latenstein CSS, Hannink G, van der Bilt JDW, SECURE trial collaborators, et al. A clinical decision tool for selection of patients with symptomatic cholelithiasis for cholecystectomy based on reduction of pain and a pain-free state following surgery. JAMA Surg. 2021;156(10):e213706. doi: 10.1001/jamasurg.2021.3706. 34379080 PMC8358816

[52] Cicala M, Habib FI, Vavassori P, et al. Outcome of endoscopic sphincterotomy in post cholecystectomy patients with sphincter of Oddi dysfunction as predicted by manometry and quantitative choledochoscintigraphy. Gut. 2002;50(5):665–668. doi: 10.1136/gut.50.5.665. 11950813 PMC1773209

[53] Judd S, Miller L, Antaki F. Symptomatic calculi in a remnant gallbladder: a rare cause of post-cholecystectomy syndrome and biliary pancreatitis. Endoscopy. 2014;46(Suppl 1 UCTN):E67–E68. doi: 10.1055/s-0033-1359193. 24523189

[54] Rogy MA, Függer R, Herbst F, et al. Reoperation after cholecystectomy. The role of the cystic duct stump. HPB Surg. 1991;4(2):129–135. doi: 10.1155/1991/57017. 1931779 PMC2443006

